# Smokeless Tobacco Initiation, Use, and Cessation in South Asia: A Qualitative Assessment

**DOI:** 10.1093/ntr/ntab065

**Published:** 2021-04-12

**Authors:** Faraz Siddiqui, Ray Croucher, Fayaz Ahmad, Zarak Ahmed, Roshani Babu, Linda Bauld, Fariza Fieroze, Rumana Huque, Ian Kellar, Anuj Kumar, Silwa Lina, Maira Mubashir, Suzanne Tanya Nethan, Narjis Rizvi, Kamran Siddiqi, Prashant Kumar Singh, Heather Thomson, Cath Jackson

**Affiliations:** 1 Department of Health Sciences, University of York, Heslington, York, UK; 2 IPH&SS Khyber Medical University, Peshawar, Pakistan; 3 Department of Community Health Sciences, Aga Khan University, Karachi, Pakistan; 4 Indian Council of Medical Research-National Institute of Cancer Prevention and Research, Noida, Uttar Pradesh, India; 5 Usher Institute, Old Medical School, University of Edinburgh, Edinburgh, UK; 6 ARK Foundation, Gulshan-2, Dhaka, Bangladesh; 7 School of Psychology, Lifton Place, University of Leeds, Leeds, West Yorkshire, UK; 8 Department of Health Sciences and Hull York Medial School, University of York, Heslington, York, UK; 9 Adults and Health Directorate, Leeds City Council, Leeds, UK; 10 Valid Research Ltd, Sandown House, West Yorkshire, UK

## Abstract

**Introduction:**

Smokeless tobacco (ST) is a significant South Asian public health problem. This paper reports a qualitative study of a sample of South Asian ST users.

**Methods:**

Interviews, using a piloted topic guide, with 33 consenting, urban dwelling adult ST users explored their ST initiation, continued use, and cessation attempts. Framework data analysis was used to analyze country specific data before a thematic cross-country synthesis was completed.

**Results:**

Participants reported long-term ST use and high dependency. All reported strong cessation motivation and multiple failed attempts because of ease of purchasing ST, tobacco dependency, and lack of institutional support.

**Conclusions:**

Interventions to support cessation attempts among consumers of South Asian ST products should address the multiple challenges of developing an integrated ST policy, including cessation services.

**Implications:**

This study provides detailed understanding of the barriers and drivers to ST initiation, use, and cessation for users in Bangladesh, India, and Pakistan. It is the first study to directly compare these three countries. The insight was then used to adapt an existing behavioral support intervention for ST cessation for testing in these countries.

## Introduction

Smokeless tobacco (ST) are noncombustible tobacco products that are chewed, snorted through the nose or placed in the oral cavity.^1^ Consumed by more than 300 million people in at least 127 countries, ST in 2017 is estimated to have caused over 90 000 deaths due to oral, pharynx, and esophageal cancers and a loss of 2.5 million disability adjusted life years.^2^ ST use also correlates with increased cardiovascular mortality risk and poor pregnancy outcomes.^2,3^ More than 85% of this disease burden concentrates in South and South East Asia.^2^

The WHO Framework Convention on Tobacco Control (FCTC)^4^ proposes a range of measures to reduce the consumption of tobacco products, including tobacco dependence treatment. Implementation of FCTC measures for ST products in general is limited,^5^ particularly with regard to cessation. Barriers exist,^6^ with many countries lacking policy and appropriate quit services.^7^ Adaptations are acknowledged as needed.^8–12^

Data informing the process of developing appropriate interventions to support cessation attempts among people consuming South Asian ST products are lacking. This article reports the results of a qualitative study of a sample of South Asian ST users used to inform the adaptation of a behavioral support intervention for ST cessation.^13^

## Methods

South Asian ST users participated in a qualitative interview study of their ST initiation, continued use and cessation attempts. Ethical approvals were granted by the Health Sciences Research Governance Committee at the University of York, Bangladesh Medical Research Council (BMRC/NREC/2016–2019/961), National Institute of Cancer Prevention and Research (NICPR) Institutional Ethics Committee (NICPR/116/DIR/Ethical/2018/02), and the National Bioethics Committee, Pakistan (4–87/NBC-355/19/1695).

Urban settings in Bangladesh, India, and Pakistan were used and 10–12 per country, exclusive (nonsmoking), daily (for the past 6 months or at least 25 days in the past month) adult ST users were interviewed. Purposive sampling incorporated both sexes, varied education levels, and users of various ST products. Participants were recruited from a primary care clinic, through local social workers, and community networks. Identified potential participants received study information and gave permission to share contact details before being contacted to arrange an interview.

Face-to-face interviews were conducted in local languages by trained and mentored country research teams in locations ensuring privacy. Online methods and analysis training was delivered by an experienced UK based qualitative researcher (CJ). Before interview start, the researcher discussed the study information sheet and secured participant consent. Participants marked or initialed the item(s) to which they consented. To ensure consistency, a topic guide was developed and piloted in all settings. Changes created better contextualization of questions, a streamlined order, and improved clarity.

The audio-recorded interviews were transcribed verbatim, checked for accuracy by the interviewers, and translated into English. Framework data analysis^14^ was conducted and findings collated for each country. A thematic cross-country synthesis and interpretation was undertaken, and illustrative quotations (Supplementary Material 1) identified. The results were reviewed by all researchers.

## Results

Thirty-three ST users were interviewed between January and August 2019 (Table 1). Interviews lasted between 24 and 83 minutes.

**Table 1.** Participant characteristics

		Bangladesh		India	Pakistan	
		Dhaka	Rangpur	Noida	Karachi	Peshawar
Gender	Male	4	2	7	4	4
	Female	3	2	5	2	0
Age	Up to 29 yr	0	0	3	1	0
	30–39 yr	0	2	3	2	1
	40–49 yr	3	1	4	3	0
	50–59 yr	1	1	0	0	2
	60 yr and above	3	0	2	0	1
Marital status	Married	4	4	10	5	4
	Single	0	0	0	1	0
	Widowed	3	0	1	0	0
	Did not report	0	0	1	0	0
Education	No formal education	0	1	1	1	1
	Primary	2	1	3	1	1
	Secondary	4	2	6	3	1
	Higher and above	1	0	1	0	1
	Did not report	1	0	0	0	0

### Smokeless Tobacco Initiation and Use Routines

Length of ST use varied by gender and country, from 1.5 to 6 years for Indian women up to 45 years in Bangladeshi men. Pakistani participants had all used ST for at least 10 years. Types of ST used varied by country. In Bangladesh *paan* with *zarda* was commonly reported whilst *guthka* and *khaini* were preferred by Indian respondents. Regional variation was observed in Pakistan, with *naswar* used in Peshawar and *guthka* in Karachi. Initiation triggers included curiosity, observation of others’ use or replacing behaviors such as smoking (Quotes 1 and 2, Supplementary Material 1).

In Bangladesh and India, women reported lower consumption frequencies than men (4–8 times per day compared with 15–40 in Bangladesh; 2–3 times per day compared with 7–8 in India). In Pakistan, frequency ranged from 5–6 to 25–30 times per day. First daily intake was integrated into early morning routines (Quote 3, Supplementary Material 1), while later use might be solitary or with work colleagues, friends and family (Quote 4, Supplementary Material 1). Higher consumption was associated with social gatherings (Quote 5, Supplementary Material 1) with a minority from all three countries describing use at weddings where nonusers were also present.

ST products were widely available in all three countries. Participants could buy ST throughout the day, with products being sold at stores, market stalls, tea stalls and vending carts, either close to home or their workplace (Quote 6, Supplementary Material 1). ST was affordable (Quote 7, Supplementary Material 1) with costs mainly described as insignificant. A Bangladeshi man reported his ST use impacted on his ability to provide for his family’s needs, while an Indian woman missed a meal to purchase ST which enabled her to continue working (Quote 8, Supplementary Material 1).

Knowledge levels of ST product content varied. Some participants offered detailed descriptions whilst others identified only one or two ingredients. Different ingredient combinations of tobacco, ash, *chuna*, supari/betel nut, natural flavorings (e.g., lime), sweeteners (e.g., sweet syrup), and chemicals were reported. Men typically had better knowledge through observation of product preparation or recognition of contents through smell and taste.

There was low awareness of ST systemic health risks. Participants might reflect on their own health, reporting headaches, chest pain, breathing difficulties, digestion problems, and feeling weak (Quote 9, Supplementary Material 1). While many noted that use during pregnancy posed risks for the unborn child some women, from all three countries, reported their using ST during pregnancy without apparent adverse effects. The oral health risks were described as blackened teeth, halitosis, gum disease, mouth ulcers, oral cancers, cuts, and thinning skin in the mouth. A Pakistani man and an Indian woman thought risks could be mitigated by mouth rinsing after use. Knowledge of health risk and ingredients likely came from health warnings in the media whilst for some their doctor had advised against ongoing use due to personal risk (Quote 10, Supplementary Material 1).

ST reportedly provided the benefits of pleasure, improved physical or mental vigor or therapeutic effects. While some reported enjoying the taste or chewing sensation, for others ST supported everyday functioning, assisting the fulfillment of work duties by increasing mental acuity, energy and physical strength or relieving nausea (Quote 11, Supplementary Material 1). ST was perceived to have positive medicinal effects on a range of existing health problems, relieving constipation, toothache, headaches, tension, insomnia, and agitation (Quote 12, Supplementary Material 1).

A minority of participants also described the perceived negative health impacts of not using ST. These could be a reduced ability to work, feelings of malaise or imbalance, physical symptoms such as stomach problems, dizziness, and seizures, or mental health problems such as agitation and aggression (Quote 13, Supplementary Material 1). Participants did not believe that they had sufficient willpower to succeed (Quote 14, Supplementary Material 1).

### Smokeless Tobacco Cessation

People’s strong wishes to quit ST was striking (Quote 15, Supplementary Material 1). Three quarters of participants had attempted ST cessation, with attempts ranging from one to 20. Most attempts were of short duration (Quote 16, Supplementary Material 1). No participants had sought cessation support when trying to quit, even after following medical advice, and most perceived doctors and other health professionals as best placed to support cessation. Multiple alternative strategies had been used in cessation attempts, including willpower, not buying products, throwing products away, delaying use to later in daily routines, avoiding consumption and replacement with alternatives like sweet *challia* (betel nut) or fennel seeds. Strong dependency feelings rendered these behavioral regulation strategies ineffective (Quotes 17, 18, Supplementary Material 1).

Cessation might be associated with fears of disapproval and stigmatization. Many reported chastisements by family (Quote 19, Supplementary Material 1) or work supervisors. Disapproval and quit requests led to concealment of use (Quote 20, Supplementary Material 1). Religious beliefs might also drive quit attempts. Some Pakistani and Bangladeshi men reported reductions in ST use during Ramadan and suggested religious leaders could encourage quitting. (Quote 21, Supplementary Material 1).

Most participants acknowledged possible adverse health consequences from ongoing use although some in Pakistan and India did not believe they would become ill. An Indian woman believed only people who were addicted or already unwell would be at risk of harm (Quote 22, Supplementary Material 1). Cessation intentions were impeded by positive physical sensations from ST use and negative physical withdrawal effects. Half the Bangladeshi and many Pakistani participants reported that whilst motivated by better health they felt addicted and powerless to stop.

Fears of becoming ill, associated treatment costs and of dying were reported (Quote 23, Supplementary Material 1). Participants also recognized positive drivers for cessation, including improved oral health, providing a role model for younger generations (Quote 24, Supplementary Material 1) and financial benefits. These were insufficient to support successful cessation.

## Discussion

The disease burdens of ST use are concentrated in in South and South East Asia.^2^ This is the first qualitative needs assessment synthesizing the views of ST users across South Asia about their ST behaviors. The results would inform the adaptation of a ST cessation behavioral intervention.^13^ The participant accounts confirm previously reported quantitative findings of long-term personal use and high dependency typical of South Asian ST users.^9,15–18^ This gives confidence that data saturation was achieved and that the findings are generalizable.^14^

New data has emerged with respect to ST cessation. All reported strong cessation motivation but many failed attempts because of ease of purchasing ST, tobacco dependency and lack of institutional support. ST use among South Asians has been reported as culturally acceptable with strong social foundations.^9^ Our participants reported a more nuanced role of significant others in their ST use. Whilst having ST using friends, family and colleagues encouraged continued personal use, participants also described discouragement from younger family members and work supervisors which created a pressure to quit, stigmatizing public ST use and encouraging lying about behavior.

Strengths and limitations of this study should be noted. We recruited men and women in three countries, across education levels, who used a variety of ST products and offered a diversity of views about their ST use. A key limitation was the failure to recruit women in one Pakistani location (Peshawar), reported to be because female ST use was considered culturally unacceptable. Second, we recruited from urban locations alone and acknowledge that ST use in rural areas may be more prevalent.^15–17^ Further research should address this study’s limitations.

Discouraging ongoing South Asian ST use requires population-level interventions to tackle opportunity factors, such as legislation, price increases and advertising bans,^10^ in addition to individual cessation support. This study suggests that implementation of policy measures and services for ST cessation is limited.^5^ Most South Asian countries lack policy, including the provision of services in which appropriately adapted behavioral resources are embedded, to help in ST cessation.

In conclusion, these South Asian ST users were highly motivated to attempt cessation yet were persistently unsuccessful because of socioenvironmental factors encouraging ST initiation, persistent drivers to continue ST consumption and lack of formal cessation resources and support. Initiatives should address these challenges in developing an integrated ST control policy, which includes cessation support for individual ST users.

## Supplementary Material

A Contributorship Form detailing each author’s specific involvement with this content, as well as any supplementary data, are available online at https://academic.oup.com/ntr.

ntab065_suppl_Supplementary_Material

ntab065_suppl_Supplementary_Taxonomy_Form
