# Supplementary material for: Smokeless Tobacco Initiation, Use, and Cessation in South Asia: A Qualitative Assessment
Source: Nicotine Tob Res. 2021 Apr 12;23(10):1801–4. doi: 10.1093/ntr/ntab065 (PMC8521714; doi:10.1093/ntr/ntab065)
Supplement: ntab065_suppl_Supplementary_Material [file ntab065_suppl_supplementary_material.docx]

**Supplementary material 1: Illustrative quotations: ST initiation, use and cessation**

| 1 | “*Like, if someone is eating it, so I see them and wonder how it would feel to eat it. My neighbours used to eat, so I learned by watching them.*”  (I_F_10: Female, Noida, 27 years, married, primary education) |
| --- | --- |
| 2 | "Well I wanted to stop smoking. Somebody told me to switch over to naswar and then slowly reduce it. Unfortunately, I am still stuck with it."  (PP-M-4: Male, Peshawar, 51 years, married, postgraduate education) |
| 3 | "*When my eyes open in the morning, I pick up packet of naswar already placed under my pillow, put a pallet in my mouth and after that I go to the washroom*."  (PP-M-2: Male, Peshawar, 53 years, married, primary education) |
| 4 | “*I use it alone at home and as well as when some close people like my friends or relatives come to visit. But I only have it with people who are users themselves."*  (PK_F_1: Female, Karachi, 42 years, married, primary education) |
| 5 | "You sit in a gathering where your friends insist you to use it, you would refuse once or twice, but then they would give justifications or motivations like; come on it’s just a “ball or pallet of naswar” it isn’t poison that will kill you."  (PP_M_1: Male, Peshawar, 35 years, married, secondary education) |
| 6 | *“You get it every five steps. You can get it from anywhere, it’s available everywhere."*  (I_M_7: Male, Noida, 48 years, married, secondary education) |
| 7 | *“Just go out and walk down any street, you will see shops upon shops selling this stuff. It costs so little everyone can afford it: women, men, even children.”*  (PK_M_1: Male, Karachi, 30 years, married, no education) |
| 8 | *“We are living in so much poverty, but still we are having food. So, I will think that I will skip a meal, because when a person is not feeling good health-wise, he won’t be able to work.“*  (I_F_14: Female, Noida, 25 years, widowed, education not reported) |
| 9 | *“I get a tingling sensation in my hands and feet. If I don't have it [ST], I have nausea. It has affected my blood. I am not as strong as I was before. I have also started having breathing problems. The more you have it, the more problems you’ll have.”*  (PK_F_2: Female, Karachi, 40 years, married, secondary education) |
| 10 | "*When in such programmes [TV adverts] it is shown that because of consuming tobacco people get cancer and consumption of tobacco is compared to consumption of poison*."  (B_M_1_R: Male, Rangpur, 36 years, married, primary education) |
| 11 | *“I have it periodically throughout the day, every time I feel like my energy is falling, I will have some."*  (PK_M_3: Male, Karachi, 38 years, married, secondary education) |
| 12 | *“There was pain in my teeth, so my grandmother said, why are you sitting like this? I said my teeth are paining. She gave it to me, and I didn’t have the knowledge about it. I rubbed it on all the teeth.”*  (I_F_14: Female, Noida, 25 years, widowed, education not reported) |
| 13 | "*I used to feel a little bit irritated and agitated. I thought, I was having some bad things happening to me. I had a fight with my wife and thought that I am abnormally agitated. So, I thought, starting it [ST] again will make my mood little bit better and my agitation may reduce."*  (PP_M_4: Male, Peshawar, 51 years, married, further education) |
| 14 | *“These [ST] are injurious to health and can cause cancer, oral ill health and cuts inside the mouth. But once you are addicted to it you can’t stop so you have it regardless.”*  (PK_F_2: Female, Karachi, 40 years, married, secondary education) |
| 15 | "*I wish I could quit using naswar. I am trying my best because it is a harmful addiction. It causes damage to teeth, stomach and gums as well.”*  (PP_M_2: Male, Peshawar, 53 years, married, primary education) |
| 16 | "*I didn’t even last one day. I had to have paan. I thought that I would not buy them ahead of time anymore. I tried to quit many times. But I never succeeded. I would last for maximum of one day. By noon of the following day I had to have paan*."  (BD_M_3: Male, Dhaka, 55 years old, married, primary education) |
| 17 | "*I take the box and keep on telling myself, “Not now. I will take it later. I am trying to check my impulses. If I feel like using, I try to take it ten or twenty minutes or half an hour later*."  (BD_M_1: Male, Dhaka, 60 years, married, secondary education) |
| 18 | *“I thought that maybe I should change to sweet challia so that I can break the habit. I stopped for a few days but started again.”*  (PK_M_Pilot: Male, Karachi, 25 years, single, secondary education) |
| 19 | "*Sometimes my son when I hug him. it is the bad smell coming from my mouth that he complains about. I am thinking that it is the right time to quit*.”  (PP_M_4: Male, Peshawar, 51 years, married, further education) |
| 20 | "*I eat it by stealing. Whenever anyone says, 'Do not eat flavoured tobacco powder, you will die.' Or my grandchildren say, 'Please do not eat it.' or my son would say, 'I will bury you in a betel leaf garden.' I say, 'No, my son, I do not eat it.' But still, I eat it secretly*."  (B_F_2_D: Female, Dhaka, 70-75 years, widowed, no education) |
| 21 | "*In last Ramadan when I was in Umrah [a pilgrimage], my use of snuff was significantly reduced because I used to take it late at night after doing all my prayers. So, on the way back I was thinking on it that if I can maintain two doses in 24 hours there is a chance of quitting*.”  (PP_M_4: Male, Peshawar, 51 years, married, further education) |
| 22 | "*It will be harmful for someone who is addicted to it but for me it is beneficial. Someone who uses it when he has a disease, it will be harmful for him, but for me it is beneficial*."  (I_F_14: Female, Noida, 25 years, widowed, education not reported) |
| 23 | "*I feel very bad then because one day I might also become a cancer patient. I remain in fear that I might become a cancer patient*."  (B_M_1_R: Male, Rangpur, 36 years, married, primary education) |
| 24 | “*Whenever I have snuff and smell is coming from my mouth so naturally if I hug them [children], and get close to them they don’t feel good but don’t say anything, but if I’m not using, they will be very happy - my mother, my wife and my kids*."  (PP_M_4: Male, Peshawar, 51 years, married, further education) |
